# A lectin of a non-invasive apple snail as an egg defense against predation alters the rat gut morphophysiology

**DOI:** 10.1371/journal.pone.0198361

**Published:** 2018-06-01

**Authors:** Santiago Ituarte, Tabata Romina Brola, Patricia Elena Fernández, Huawei Mu, Jian-Wen Qiu, Horacio Heras, Marcos Sebastián Dreon

**Affiliations:** 1 Instituto de Investigaciones Bioquímicas de La Plata (INIBIOLP), Universidad Nacional de La Plata (UNLP)–CONICET, La Plata, Argentina; 2 Instituto de Patología B. Epstein, Cátedra de Patología General Veterinaria, Facultad Ciencias Veterinarias, UNLP, La Plata, Argentina; 3 Department of Biology, Hong Kong Baptist University, Hong Kong, China; 4 Cátedra de Química Biológica, Facultad de Ciencias Naturales y Museo, UNLP, La Plata, Argentina; 5 Cátedra de Bioquímica y Biología Molecular, Facultad de Ciencias Médicas, UNLP, La Plata, Argentina; Duke University Marine Laboratory, UNITED STATES

## Abstract

The eggs of the freshwater *Pomacea* apple snails develop above the water level, exposed to varied physical and biological stressors. Their high hatching success seems to be linked to their proteins or perivitellins, which surround the developing embryo providing nutrients, sunscreens and varied defenses. The defensive mechanism has been unveiled in *P*. *canaliculata* and *P*. *maculata* eggs, where their major perivitellins are pigmented, non-digestible and provide a warning coloration while another perivitellin acts as a toxin. In *P*. *scalaris*, a species sympatric to the former, the defense strategy seems different, since no toxin was found and the major perivitellin, PsSC, while also colored and non-digestible, is a carbohydrate-binding protein. In this study we examine the structure and function of PsSC by sequencing its subunits, characterizing its carbohydrate binding profile and evaluating its effect on gut cells. Whereas cDNA sequencing and database search showed no lectin domain, glycan array carbohydrate binding profile revealed a strong specificity for glycosphingolipids and ABO group antigens. Moreover, PsSC agglutinated bacteria in a dose-dependent manner. Inspired on the defensive properties of seed lectins we evaluated the effects of PsSC on intestinal cells both *in vitro* (Caco-2 and IEC-6 cells) and in the gastrointestinal tract of rats. PsSC binds to Caco-2 cell membranes without reducing its viability, while a PsSC-containing diet temporarily induces large epithelium alterations and an increased absorptive surface. Based on these results, we propose that PsSC is involved in embryo defenses by altering the gut morphophysiology of potential predators, a convergent role to plant defensive lectins.

## Introduction

The reproductive strategy of the freshwater *Pomacea* snails involves laying colored egg clutches above the water line, exposing them to a set of environmental stressors including direct sunlight, high temperatures, and diverse predators such as insects, birds and rodents [1,2]. These cleidoic eggs have a high hatching rate [3] which in some species of the genus, such as *P*. *canaliculata* and *P*. *maculata*, enhances its biological potential as invasives [4,5]. Notably, both species were introduced to Southeast Asia in the 1980’s, and rapidly became a serious socio-economic problem as a rice crop pest, a voracious herbivore for aquatic vegetation and a vector of a human parasite [5–7].

The eggs of these snails are small (2.5 mm), rounded, covered with a calcareous capsule and glued to each other in masses of 200–1500 eggs. A disctinctive characteristic of these eggs is coloration, which varies from bright pink in *P*. *canaliculata* and *P*. *maculata* to pale orange in *P*. *scalaris* [2]. The oocytes are surrounded by a perivitelline fluid (PVF) mainly composed by proteins and polysaccharides [8,9]. Biochemical and physiological evidence indicates that *Pomacea* egg proteins, hereafter perivitellins, in addition to their role as energetic and structural sources, are involved in embryo adaptation to the adverse environmental conditions of development [10–12]. Remarkably, *P*. *canaliculata* eggs have no reported predators in the native area of this species; recent studies showed that they are well defended by multifunctional perivitellins, including the neurotoxin PcPV2, which is lethal to mice [13] and the antinutritive carotenoprotein PcOvo, which can reduce rat growth rate and provide a warning signal (aposematic coloration) [14]. These proteins showed high structural stability in a wide range of temperature and pH values. Moreover, both perivitellins are resistant to simulated gastrointestinal digestion, thus being able to reach the intestinal tract of a potential egg predator in a biologically active conformation [14,15]. Comparable findings were reported in *P*. *maculata* eggs, where their major perivitellins, PmPV1 and PmPV2, showed similar structural and functional properties as PcOvo and PcPV2, respectively [12,16,17].

In contrast, *Pomacea scalaris*, a species sympatric to *P*. *canaliculata* and *P*. *maculata*, showed different properties in its major egg protein: PsSC. This perivitellin is an oligomeric carotenoprotein of 380 kDa which, despite sharing structural and functional properties with PcOvo and PmPV1 [11,18], has a distinctive feature: a strong lectin activity, agglutinating rabbit and, to a lesser extent, human erythrocytes [19].

The presence of highly stable carotenoproteins as the most abundant proteins in the egg PVF appears as a common characteristic in *Pomacea* snails, possibly a key biochemical adaptation to aerial egg development [10]. The presence of a carotenoprotein with lectin activity in a *Pomacea* egg was an unexpected finding, showing that within the genus carotenoproteins exhibit structural and/or functional divergence. Lectins are carbohydrate binding proteins, found in animals and plants, able to specifically recognize oligosaccharide structures. They are known to play essential roles in the innate immune system of invertebrates recognizing microbe-associated molecular patterns (MAMPs) of invading microorganisms. Therefore, a protective role against microbial invasions is generally assumed for egg lectins [20], though this assumption has not been tested for the eggs of many animals. Finally, considering PsSC resistance to digestive proteases [19], it is possible that this perivitellin is also involved in egg defense against predation through a mechanism similar to the one thoroughly studied for plant lectins [21].

The aim of this work was to shed light on the role of PsSC in egg defenses. We determined its primary structure and characterized its glycan binding specificity. We also tested its antimicrobial activity and explored the effect of oral administration of a PsSC–containing diet on the digestive tract of rats as a model of potential egg predator. Finally, we investigated *in vitro*, the specific interaction of PsSC with digestive epithelium surfaces and its cytotoxic effect on enterocyte cell lines.

## Materials and methods

### Ethics statement

Animal studies were carried out in accordance with the Guide for the Care and Use of Laboratory Animals [22] and were approved by the Institutional Animal Care and Use Committee (IACUC) of the School of Medicine, UNLP (Permit No. P01-01-2016). Our research adheres to the legislation of the Argentinean provincial Wildlife Hunting Law (Ley 5786, Art. 2).

### Isolation and purification of PsSC

Purification of PsSC was carried out as previously described [11] from newly-laid egg clutches collected from the Regatas pond (Ciudad Autónoma de Buenos Aires, Argentina). Briefly, egg homogenates were centrifuged sequentially at 10,000 g for 30 min, and 100,000 g for 60 min. The obtained supernatant was fractionated by ultracentrifugation on a NaBr density gradient. Finally, fractions containing PsSC were purified by size exclusion chromatography. The purity of the protein obtained from a single chromatographic peak was checked by native PAGE performed in a Mini-Protean III System (Bio Rad Laboratories, Inc.), and protein content was determined by the method of Bradford [23].

### Primary structure and subunit sequences analysis

Mass spectrometry analysis of purified PsSC was conducted to obtain amino acid sequences of the tryptic peptides. The obtained sequences, together with one N-terminal sequence previously reported [19], were used to search the cDNA sequences of the different PsSC subunits in the *P*. *scalaris* albumen gland transcriptome [24]. The signal peptide cleavage sites in the translated amino acid sequences of PsSC were predicted using the SignalP 4.1 server [25]. The theoretical molecular weight and isoelectric point of each mature subunit were estimated using the ProtParam tool-Expasy server [26]. Finally, potential phosphorylation and glycosylation sites were predicted with DISPHOS 1.3 [27,28] and NetNGlyc 1.0 server, respectively.

Putative PsSC subunits were determined by search using the BLAST program (National Library of Medicine). Sequences were aligned using MAFFT 7 [29] and phylogenetic trees were constructed by the maximum likelihood method with bootstrapping analysis using MEGA version 6 [30].

### Glycan array

Glycan binding specificity of PsSC was determined at the Core H of the Consortium for Functional Glycomics (http://www.functionalglycomics.org, Emory University, Atlanta, GA, USA). In order to detect primary binding of PsSC to glycans, the protein was fluorescently labeled using the Alexa Fluor 488 Protein Labeling kit (Invitrogen, Life Technologies-Molecular Probes) according to the manufacturer’s instructions. Protein concentration and the degree of labeling were determined spectrophotometrically. Fluorescently labeled PsSC was assayed on a glycan array which comprised 610 glycan targets (version 5.1) and the data analyzed as previously described by Smith et al. [31]. In order to eliminate false hits containing a single very high point, the highest and lowest points from a set of six replicates were removed and the four remaining values were averaged. Oligosaccharides with high %CV were excluded.

### Antimicrobial activity assays

We tested the antimicrobial activity of PsSC on *Escherichia coli* (BL-21) and *Staphylococcus pseudointermedius* cultures in solid and liquid media. In each plate, 50 μL of culture was spread onto LB/agar in triplicates, and 20 min later 10 μL drops containing 20, 10 and 2 μg of PsSC were dispensed. Sterile buffer was used as negative control. The plates were incubated for 18 h at 37°C and the formation of inhibition rings was observed. In the liquid media assays, 500 μL of culture was diluted with LB in triplicates and PsSC was added to a final concentration of 0.2 g/L, with sterile PBS being used as control. Growth was measured as optical density at 600 nm.

We also tested the microbial agglutinating activity of PsSC. In the assays we incubated 40 μL of *E*. *coli* in PBS (OD_600_ = 1.2) on 96-well plates with 10 μL of serial dilutions of PsSC overnight at room temperature. Lectin activity was confirmed by competition with 20 mM GalNAc and 20 mM GlcNH2 (final concentrations), which were preincubated with PsSC prior to its addition to the well. After incubation, the plates were tilted for 30 min and observed by naked eye, additionally, the content of the well was observed under a microscope to confirm microbe agglutination.

### Binding assay to cell surface

Human colorectal adenocarcinoma (Caco-2) and rat small intestine crypt cells (IEC-6) obtained from the ATCC (Cerdalane Inc, Burlington, ON) were cultured as previously described [15]. Briefly, cells were cultured at 37°C, 5% CO_2_ and subcultured by trypsinization at 95% confluency. Passages 60 through 65 and 16 through 20, for Caco-2 and IEC-6 cells respectively, were used for the experiments. Prior to each experiment, cell viability was checked to exceed 90% by the Trypan Blue dye exclusion test.

For the PsSC binding assay, harvested cells were seeded on 24-well plates (Orange Scientific, Braine-l’Alleud, Belgium) at densities that ensured approximately 80% confluence in controls at the end of a 2-day experiment. Culture medium was replaced daily and, after 48h incubation, cells were washed twice with PBS and incubated with a PsSC as well as with an Alexa488 labeled PsSC preparation in PBS (400 μg/ml) for 1 h at 37°C. Cells were observed in an inverted fluorescence microscope (Olympus IX-71). Negative control wells were incubated with BSA as well as with an Alexa488 labeled BSA in PBS. All protein preparations used in cell culture assays were sterilized by filtration (0.22 μm). Protein labeling was done using Alexa Fluor 488 protein labeling kit (Invitrogen) following manufacturer’s directions.

### Cytotoxicity

Cytotoxicity of PsSC on Caco-2 and IEC-6 cells was evaluated using the MTT cell proliferation assay as previously described [32]. Briefly, cells were seeded on 48-well plates at densities that ensured approximately 90% confluency after 24 h. After that, 50 μL/well of a serial dilution of PsSC (3.4 mg/mL) in PBS were added and incubated at 37°C for 24 h. Control wells were prepared with 50 μL/well of PBS. After treatments, culture medium was removed and cells were incubated with fresh medium containing 0.5 g/L of MTT at 37°C for 1 h. Cells were washed three times with PBS and cell monolayers extracted with 200 μL/well of DMSO. Absorbance was recorded at 540 nm in a DTX-880 Multimode Detector (Beckman Coulter) and cell viability expressed as control percentage.

### Effect of PsSC-containing diet on rat gut epithelium

The effect of a PsSC containing diet on small intestine epithelium was evaluated using male Wistar rats from the Animal Facility Colony of the School of Medicine, UNLP (strain WKAHlHok, Hokkaido University, Japan). Based on previous results with PcOvo, a PsSC orthologue, on the Wistar rat growth [14] nine young animals (5 to 7 weeks-old) weighing 150 ± 5 g were randomly assigned to control, 48h treated group or 72 h treated group (N = 3 per group) and placed in a conditioned room with a 12:12 l:d cycle at 22 ± 1°C and 45–60% relative humidity. Treated groups were gavaged, using an animal feeding needle (Popper and Sons, Inc), with 1 mL of egg soluble fraction containing 8 mg total protein (~5 mg of PsSC) in 50 mM phosphate buffer (pH 7.4) on a daily basis for 48 h and 72 h. The control group was administered with the same amount of buffer without the PVF. After treatments, animals were euthanized by CO_2_ inhalation in a closed chamber. The first segment of the small intestine was cut, rinsed 6 times with PBS to remove food and unbound protein, and fixed in 4% phosphate buffered formalin (pH 7.0) for histological examination.

### Histology and morphological measurements

Small intestine samples were embedded in paraffin wax as previously reported [32]. Four tissue sections of each animal were stained with haematoxylin and eosin for examination of general morphology and with periodic acid Schiff (PAS) to highlight carbohydrate distribution on goblet cells.

Mucosal absorptive surface area of fifty random villi and crypts from duodenum from each animal was calculated following the method of Kisielinski [33]. In brief, the mucosa is assumed to be an iteration of a geometric mucosal unit: a cylindrical villous with rounded tip surrounded by cylindrical crypts. Surface area can be calculated with mean values of structures that define this mucosal unit: villous length, villous width, and crypt width. Finally, the mucosal-to-serosal amplification ratio (*M)* is calculated considering these three variables:

$$M=\frac{(villous\;width\times villous\;length)+{\left(\frac{villous\;width}{2}+\frac{crypt\;width}{2}\right)}^{2}-{\left(\frac{villuos\;width}{2}\right)}^{2}}{{\left(\frac{villous\;width}{2}+\frac{crypt\;width}{2}\right)}^{2}}$$

### Lectin histochemisty

To reveal changes of the glycosylation pattern, four small intestine sections of each animal were assayed employing a kit of seven biotinylated lectins (Table 1) (Lectin Biotinylated BK 1000 Kit, Vector Laboratories Inc., Carpinteria, CA, USA). Deparaffinized sections were incubated with the biotinylated lectins overnight as described elsewhere [32], incubated with Streptavidin-HRP (Vector Laboratories Inc., USA) and bound lectins visualized by 3, 3’-diaminobenzidine (Dako, Carpinteria, USA). Positively-stained cells were revealed by a dark golden brown coloration. The sections were counterstained with Mayer’s haematoxylin.

**Table 1:** Acronyms and major sugar specificities of the lectins employed in this study.

Lectin	Acronym	Major Specificity
*Concanavalina ensiformis*	ConA	α-D-Man; α-D-Glc
*Dolichos biflorus*	DBA	α-D-GalNAc
*Glycine maximus*	SBA	α–D-GalNAc; β–D-GalNAc
*Arachis hypogaea*	PNA	β-D-Gal (β 1–3) D-GalNAc
*Ricinus communis-I*	RCA-I	β-Gal
*Ulex europaeus-I*	UEA-I	α-L-Fuc
*Triticum vulgaris*	WGA	β-D-GlcNAc; NeuNAc

Major specificities according to Goldstein and Hayes [34]. Fucose (Fuc), Galactose (Gal), N-Acetyl galactosamine (GalNAc), Glucose (Glc), N-Acetyl glucosamine (GlcNAc), Mannose (Man) and Acetyl neuraminic acid / sialic acid (NeuNAc).

### Statistical analysis

Data were analyzed by one way analysis of variance (ANOVA). When p values were < 0.05, the significance between groups was estimated by the Tukey’s test.

## Results

### PsSC aminoacid sequences

**Fig 1:**
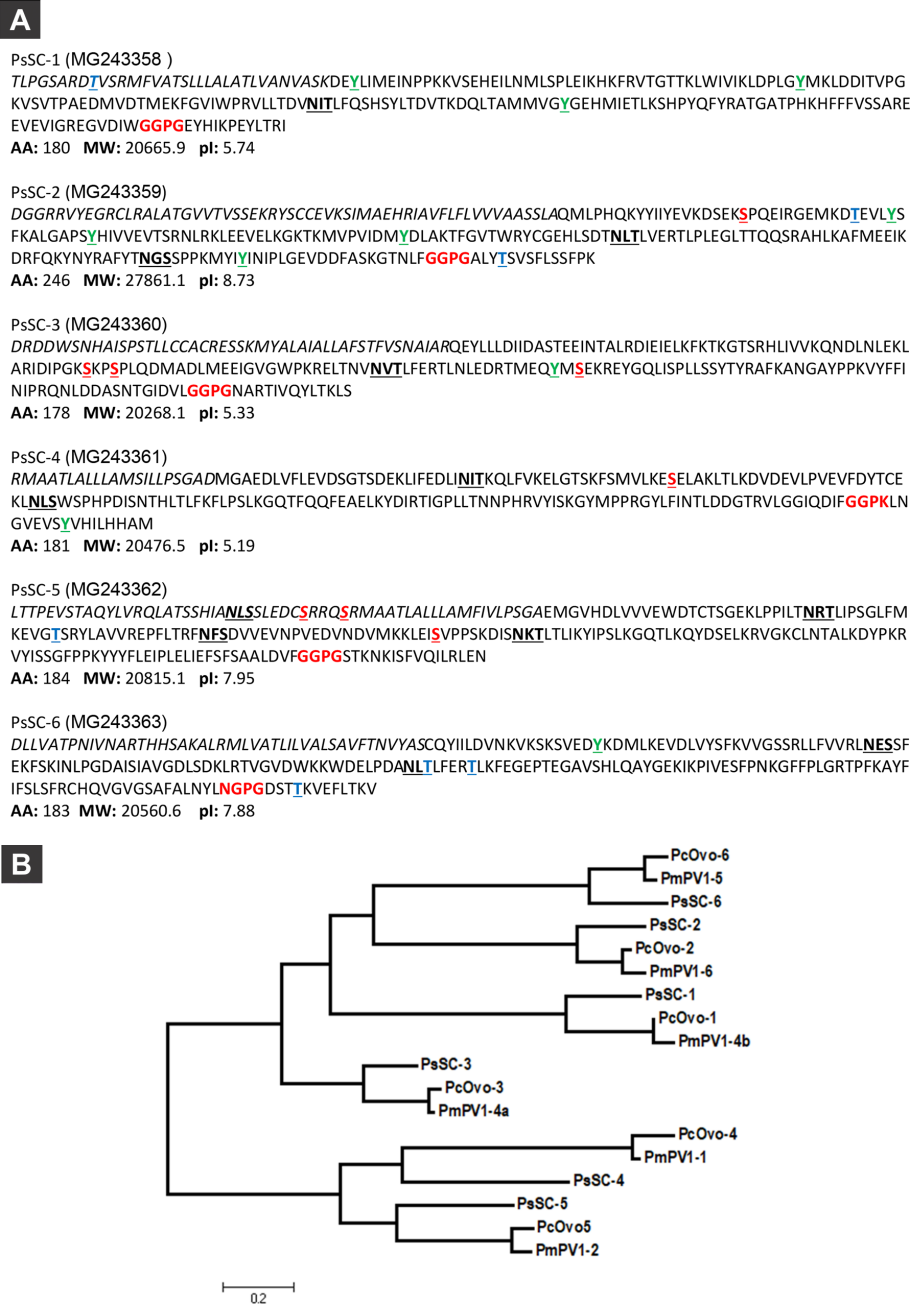
**A, Deduced amino acid sequences of PsSC subunits.** The putative signal sequences are in italics. Potential phosphorylation sites are underlined in bold green (Tyr), bold red (Ser) and bold blue (Thr). Potential N-glycosylation sites (NXS/T) are underlined and in bold. Conserved sequence in bold red. AA: number of residues, MW: molecular weight and pI: isoelectric point of the subunit mature forms. B, Phylogenetic analysis of the major perivitelins of *P*. *scalaris*, *P*. *canaliculata* and *P*. *maculata*. Tree was constructed by the Maximum Likelihood method and drawn to scale, with branch lengths measured in the number of substitutions per site. All positions containing gaps and missing data were eliminated. There were a total of 165 positions in the final dataset.

The internal amino acid sequences of purified PsSC determined by mass spectrometry analysis allowed us to obtain 6 full length cDNA sequences of its subunits from the albumen gland transcriptome of *P*. *scalaris* (unpublished). The nucleotide sequences were deposited in GenBank database (www.ncbi.nlm.nih.gov/genbank/) with accession numbers: MG243358, MG243359, MG243360, MG243361, MG243362 and MG243363. Translated amino acid sequences of the subunits (PsSC-1 to 6) are shown in Fig 1A. They all contain a signal peptide sequence and their theoretical molecular masses are around 21 kDa, except for PsSC-2 (28 kDa), which is in agreement with the MW values reported for the chemically deglycosylated PsSC subunits [1]. N-glycosylation sites (NXS/T) were predicted in the 6 subunits, being PsSC-5 the most N-glycosylated subunit with three putative sites. Regarding the presence of phosphorylation sites, in PsSC-1 subunit three Tyr associated sites were predicted; four Tyr, one Ser and two Thr associated sites were predicted for PsSC-2; one Tyr and three Ser for PsSC-3; one Tyr and one Ser for PsSC-4; one Ser and one Thr for PsSC-5, and one Tyr and three Thr for PsSC-6. Multiple sequence alignment showed a short, conserved sequence, G/N-G-P_G/K, at the C-terminal region of the 6 subunits.

### Phylogenetic analysis

The translated amino acid sequences obtained for the six PsSC subunits showed high similarity with their PcOvo orthologous subunits (40.56 to 68.78%) and with PmPV1 orthologous subunits (42.78 to 70.86%). Interestingly, sequence similarity among the 6 PsSC subunits was moderate (16.96 to 41.67%), as was previously observed in PcOvo [35] and PmPV1 [17].

Sequence alignment of PsSC subunits and their homologs showed a few conserved sites for all 18 sequences, particularly, the GGPG site previously reported on the C-terminal region of PcOvo and PmPV1 subunits.

Phylogenetic analysis of PcOvo, PmPV1 and PsSC sequences defined six different clades (Fig 1B). Notably, no sequence similarity to any known lectin could be found in any of the PsSC subunits.

### Glycan array

The assay was performed using three different protein concentrations (2, 20 and 200μg/mL) which gave similar results. Table 2 shows the 10 best ranked oligosaccharide structures recognized by PsSC. Among these, two major non-related oligosaccharide groups can be recognized: glycosphingolipids and ABO group antigens. Ganglioside-related structures showed the highest scores (GD1b>GT1b>GD1a) while the highest scores for ABO group antigens were for types 2B and 2A. The full list of oligosaccharide structures recognized by PsSC is shown in S1 Table.

**Table 2:** Main oligosaccharide structures recognized by PsSC from *Pomacea scalaris* ordered in a decreasing level of affinity.

Rank	Oligosaccharide Structure	Average RFU	%CV
1	Galb1-3GalNAcb1-4(Neu5Aca2-8Neu5Aca2-3)Galb1-4Glcb-Sp0	12556 ± 1216	10
2	Fuca1-2Galb1-3GalNAcb1-3Gala1-4Galb1-4Glcb-Sp9	8812 ± 2007	23
3	Neu5Aca2-3Galb1-3GalNAcb1-4(Neu5Aca2-8Neu5Aca2-3)Galb1-4Glcb-Sp0	8452 ± 558	7
4	Neu5Aca2-3Galb1-3GalNAcb1-3Gala1-4Galb1-4Glcb-Sp0	8067 ± 159	2
5	Neu5Aca2-3Galb1-3GalNAcb1-4(Neu5Aca2-3)Galb1-4Glcb-Sp0	7969 ± 326	4
6	GalNAca1-3GalNAcb1-3Gala1-4Galb1-4Glcb-Sp0	7468 ± 722	10
7	GalNAcb1-3Gala1-6Galb1-4Glcb-Sp8	7162 ± 450	6
8	Galb1-4GlcNAcb1-2Mana-Sp0	7157 ± 369	5
9	Galb1-3GalNAca1-3(Fuca1-2)Galb1-4GlcNAc-Sp0	7116 ± 136	2
10	Gala1-3(Fuca1-2)Galb1-4GlcNAcb1-3GalNAca-Sp14	7047 ± 346	5

Binding intensities expressed as the mean of relative fluorescence units (RFU) ± 1SD, N = 4. %CV = 100 x SD. Full data of PsSC glycan specificity is available as Supporting Information.

### Antimicrobial activity

**Fig 2:**
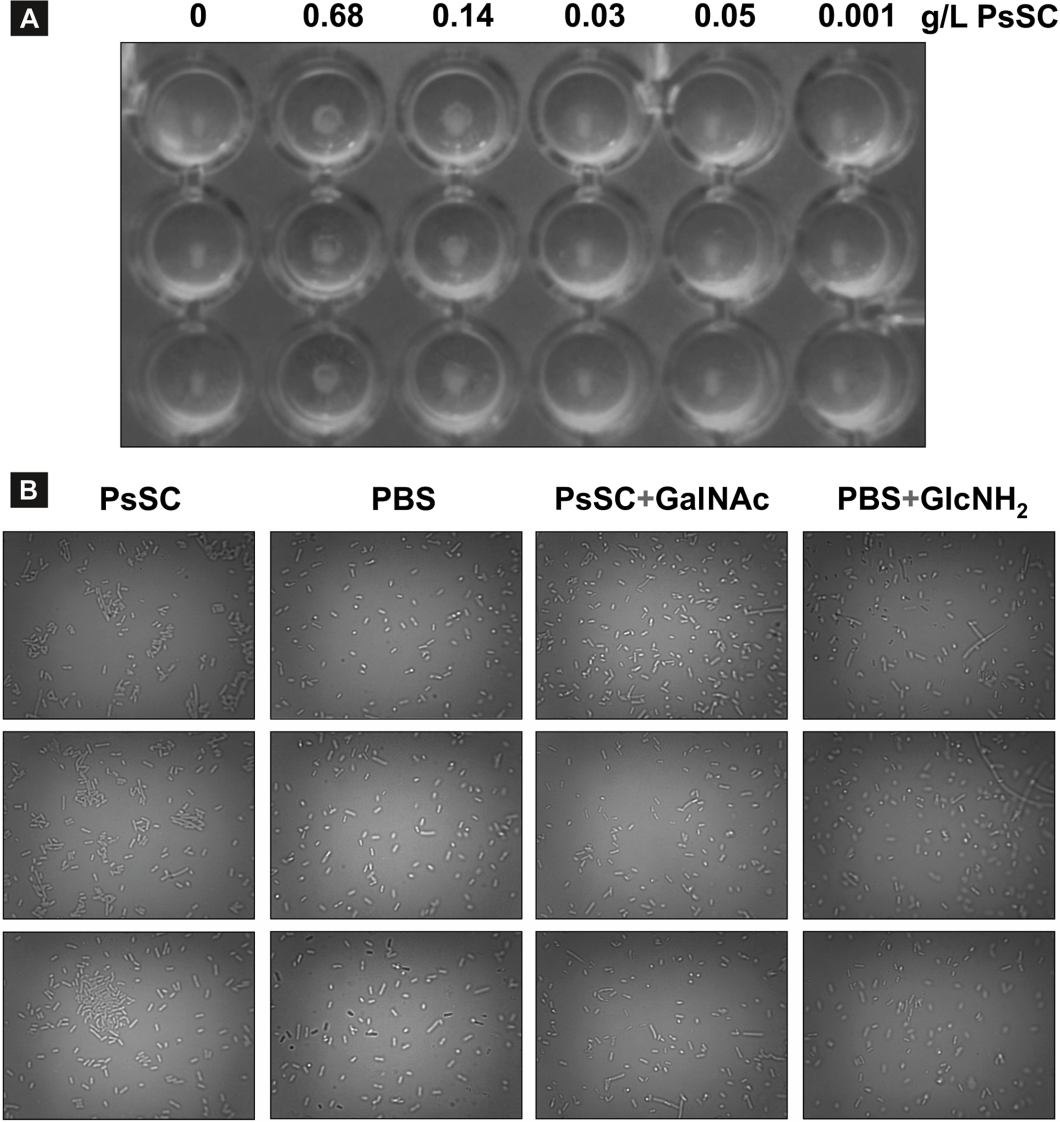
Microbial agglutinating capability of PsSC on *E*.*coli* BL21 strain. A) Rounded well plate containing aliquots of *E*. *coli* culture incubated with different PsSC concentrations. B) Inhibitory effect of GalNAc and GlcNH2 on PsSC microbe agglutinating activity.

The antimicrobial activity of PsSC was explored on Gram negative (*E*. *coli*) and Gram positive (*S*. *pseudointermedius*) bacteria. Results showed no antibacterial activity of the protein in liquid and solid media, at least under the experimental conditions employed. Nevertheless, PsSC showed agglutinating activity against *E*. *coli* in a dose-dependent manner up to 0.14 g/L of protein (Fig 2A). Lectin activity was inhibited by the presence of the monosaccharides already reported as targets of PsSC, GlcNH2 and GalNAc (Fig 2B).

### Cell surface interaction and cytotoxicity of PsSC

**Fig 3:**
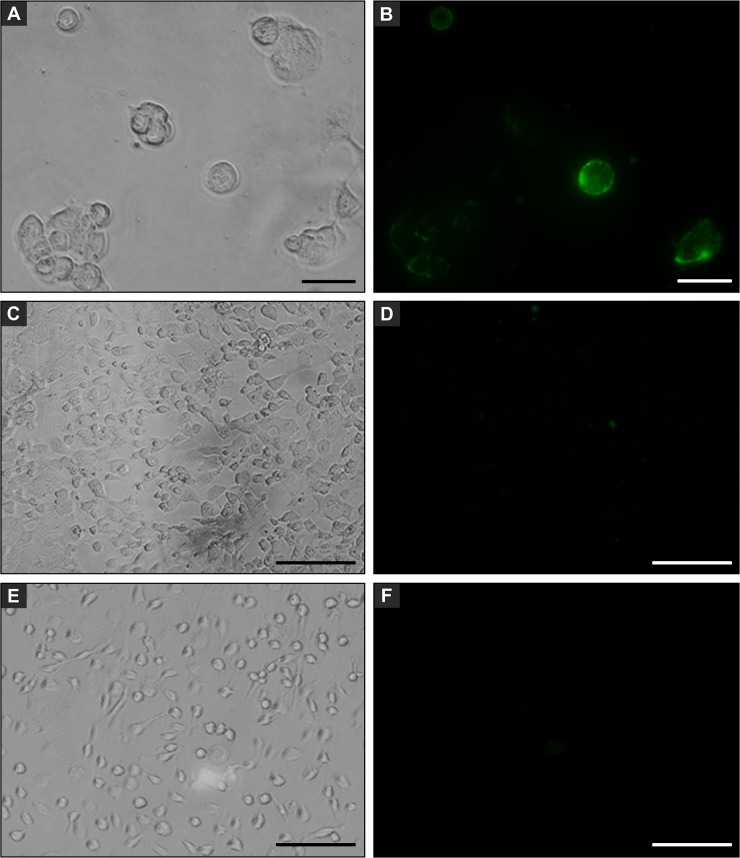
Binding of fluorescently-labeled PsSC to intestinal cells in culture. Caco-2 cell culture incubated for 1 h with Alexa-488 labeled PsSC observed by phase contrast (A) and fluorescence microscopy (B), bars: 50 μm. C and D same as A and B employing Alexa-488 labeled BSA, bars: 100 μm. E and F same as A and B employing IEC-6 cell line, bars: 100 μm.

Protein localization studies employing Alexa488-labeled PsSC showed weak but measurable fluorescence signal on Caco-2 cells plasma membrane (Fig 3A and 3B). To rule out the possibility of nonspecific protein-cell interactions, Caco-2 cells were incubated with Alexa488-labeled BSA (Fig 3C and 3D), which showed negligible binding. Assuming that BSA binding represents the maximum nonspecific interaction between Caco-2 and proteins [36], this assay indicates specific binding of PsSC to Caco-2 cell surfaces. The experiments were also performed using the IEC-6 cell line but no interaction was detected (Fig 3E and 3F). Although protein localization experiments indicate a specific interaction of PsSC with Caco-2 cell surfaces, no cytotoxic effect could be detected either on Caco-2 or IEC-6 cells (not shown).

### Effect of PsSC-containing diet on digestive epithelium

**Fig 4:**
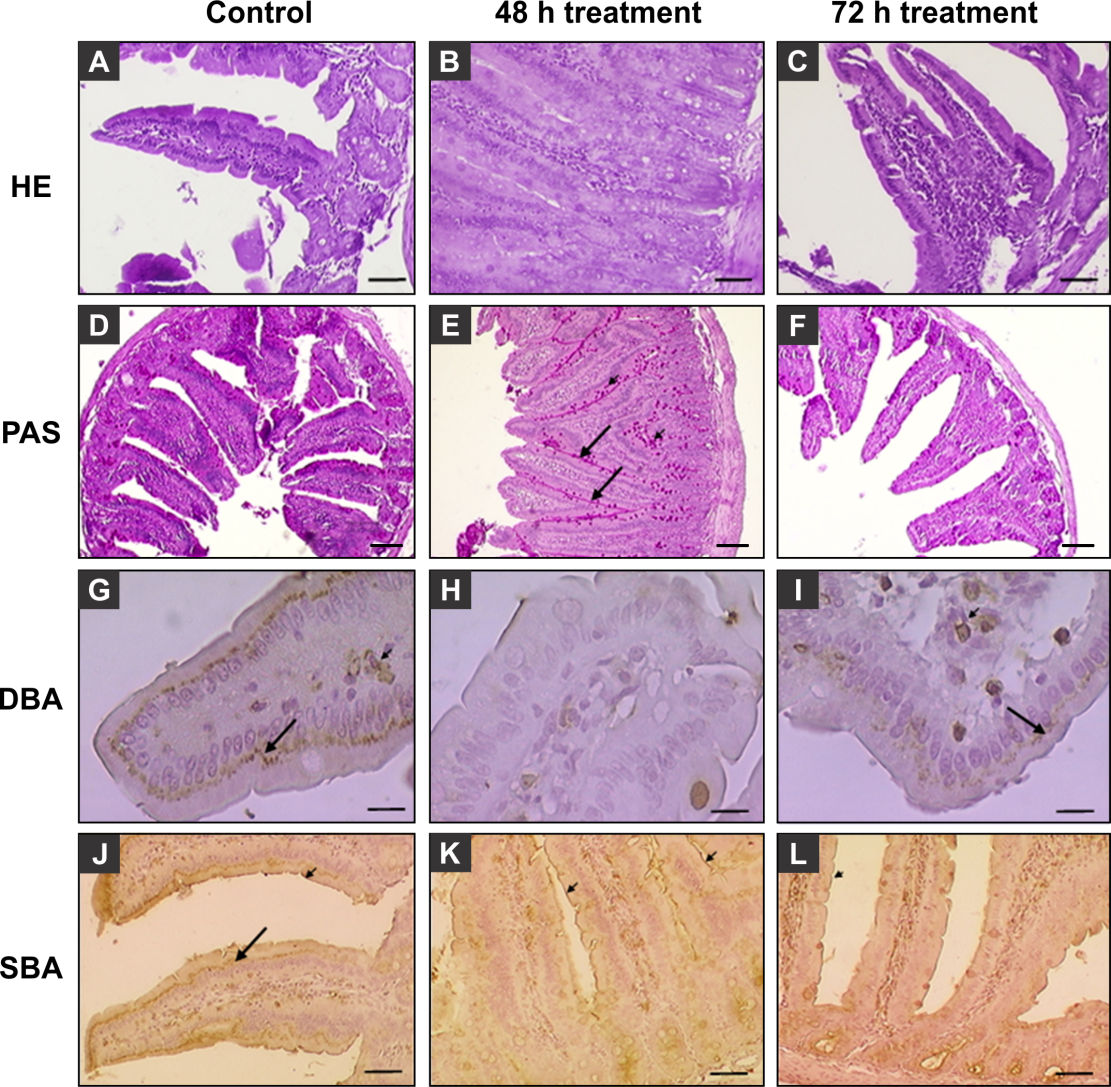
Effects of PsSC containing diet on rat small intestine morphology, glucids, and glycosylation pattern. Rats were fed with a diet without (control) or supplemented with PVF containing 8 mg protein (~ 5 mg PsSC). The duodenal portion of the small intestine was sampled after 48 and 72 h. A,D,G,J, Control; B,E,H,K 48 h treatment; C,F,I,L 72 h treatment. A,B,C: HE stain. Most villi for 48 h-treated animals were higher, narrower, more tortuous and sinuous, with some proliferation in the basal zone of the epithelia. D,E,F: PAS stain highlighting the glycocalix (arrow) and goblet cells (arrowheads) which increased after 48 h treatment. G,H,I: DBA lectin histochemistry. Arrows: supranuclear zone of the enterocyte, arrowhead corion cells of the villi. J,K,L: SBA lectin histochemistry. Arrows indicate supranuclear zone and arrowheads glycocalyx. A,B,C,J,K,L, Bar: 50 μm; D,E,F Bar: 100 μm; G,H,I: Bar: 10 μm.

Small intestine of the 48 h-treated animals showed higher, narrower, more tortuous and sinuous villi with respect to the controls, with some proliferation and the appearance of immature enterocytes in the crypts. The 72 h-treated animals showed normal crypt dimensions and general morphology of intestine (Fig 4A, 4B and 4C). PAS staining on the glycocalyx of villi and crypts in 48 h-treated animals was stronger than the controls, being less strong to moderate in 72 h-treated animals. The mucin of goblet cells showed a strong PAS stain in all groups, although treated animals showed an increased number of these cells (Fig 4D, 4E and 4F). Among the seven lectins assayed, DBA and SBA produced the most remarkable differences (Fig 4G–4L). DBA was strongly positive on the supranuclear region of enterocytes and some corion cells of control animals, while in 48 h-treated animals no binding was observed in those regions. In 72 h-treated animals the pattern was similar to control though with less intensity of staining (Fig 4G, 4H and 4I arrows). The binding of SBA lectin to enterocytes in control animals was moderate to strong on the supranuclear region and moderate at the glycocalyx (Fig 4J), while in the 48 h-treated animals stronger lectin binding was limited to the glycocalyx (Fig 4K, arrow); the 72 h-treated animals displayed a less marked lectin staining pattern. These results indicate that both DBA and SBA-binding glycans were differentially expressed on enterocytes exposed to a PsSC-containing diet for 48 h.

Finally, a significant increase in the absorptive surface of the small intestine was observed in the 48 h treated animals as compared to the control ones, while in the 72 h group, the absorptive surface reverted to normal (Table 3).

**Table 3:** Mucosal absorptive surface of small intestine of rats fed with control or PsSC-containing diet after 48 and 72 h.

	48 h	72 h
**PsSC-containing diet**	7.01 ± 2.06*	5.75 ± 1.23
**Control diet**	5.99 ± 1.11	5.99 ± 1.11

Values represent the mean ± SD of 3 rats (n = 50 cross-section/animal).

*p<0.05.

## Discussion

The presence of multifunctional, highly stable and colored carotenoproteins as the major egg proteins emerges as a general characteristic in *Pomacea* species. In this work we determined the primary structure of PsSC subunits, the most abundant soluble protein (64% total protein) in *P*. *scalaris* eggs [11]. As in PcOvo and PmPV1 oligomeric carotenoproteins [12,37], we show that PsSC oligomer is also composed of six polypeptides, with moderate sequence similarity among them, reinforcing the hypothesis that gene duplication took place early in the history of the *Pomacea* genus [35]. Interestingly, phylogenetic analysis clustered PcOvo, PmPV1 and PsSC subunit sequences in six different clades, each composed of only one subunit of each carotenoprotein. The low divergence between orthologous sequences could indicate either that after the duplication event each paralogue acquired a specific function that prevented additional variations, or that there was a benefit in having multiple copies of the same gene such that carotenoprotein synthesis can be enhanced [38]. In the case of *Pomacea* perivitellins the second scenario seems more plausible, as carotenoproteins are highly expressed during egg production [39]. The phylogenetic clustering of each one of PsSC subunit sequences with their PcOvo and PmPV1 orthologues agrees with the current species grouping, since *P*. *scalaris* was recovered as an independent (although sister) clade–the *bridgesii* clade–from that of *P*. *canaliculata* and *P*. *maculata* -the so-called *canaliculata* clade in recent phylogenetic studies [2].

Although the biological function of a protein may be predicted based on its primary structure, in the case of PsSC the six deduced amino acid sequence subunits showed no similarity with any known lectin motif. This may be due not only to the poor representation of invertebrate lectins in databases, but also to the high number and diversity of invertebrate species that comprise over 90% of all animal species. This lack of sequence identity with known lectins has also been observed for other mollusk lectins, such as the agglutinin from *Octopus maya* hemolymph [40]. Moreover, more recently, Chickalovets et al. [41] described a Gal/GalNAc-specific lectin from the mussel *Mytilus trossulus* with no sequence similarity with any known lectin, except two proteins with agglutinating activity MytiLec [42] and CGL [43] allowing the authors to describe a new lectin family.

In this work we further characterized the lectin activity of PsSC. It is noteworthy that the five oligosaccharide structures with the highest affinity contain Galβ1-3GalNAc which are present in the major core structures of vertebrate glycosphingolipids (subfamilies Lacto, Ganglio and Globo). The lectin recognized two non-related oligosaccharide structures, ABO human blood group antigens and gangliosides. This rather broad specificity suggests the presence of more than one high affinity recognition site in PsSC. This oligosaccharide recognition specificity was validated *in vitro* by PsSC binding to Caco-2 cell surfaces, since such sugar structures are well represented in this cell line [44]. Although Caco-2 viability was not affected by PsSC, oral administration of a PsSC containing diet to rats alters the gut epithelium, reversibly affecting enterocyte morphology and inducing glycocalix remodeling. The PsSC-containing diet markedly influenced enterocyte turnover inducing the appearance of immature enterocytes in the crypts of treated animals, this proliferation indicates an increase of intestine length explaining the increase in absorptive surface. This is a common mechanism to revert the loss of absorptive surface, as increasing length increases the transit time and allows digestion of food [32]. Associated with proliferation changes where also observed in the glycosylation pattern with a differential binding of DBA and SBA plant lectins. These lectins, as PsSC, have also specificity for GalNAc-containing oligosaccharides. Interestingly, these remarkable morphological and glycosylation changes tend to return to normal after 72 h treatment together with the absorptive surface, indicating an adaptation of rats overcoming the adverse effect of PsSC. Similar adaptive responses to overcome a lectin-mediated brush border disruption were also reported for plant lectins [45], suggesting that both plant and snail defensive lectins induce large but reversible changes in intestinal morphology, at least in rats. Though PmPV1, an orthologous protein of PsSC, was able to resist the mice gastrointestinal tract without structural perturbations [17], the effect of different potential egg predator microbiotas could play an important role in the anti-nutritive effects of PsSC.

It is well established that mollusk lectins are part of the innate immune system recognizing microbe-associated molecular patterns (MAMP) [46]. Moreover, mollusk lectins, such as the sea mussel *Crenomytilus grayanus* lectin (CGL), are involved not only in the recognition, but also in the clearance of the invading microbes [43,47]. Although the physiological implications of the presence of a lectin inside an egg may have different interpretations, the ability of PsSC to agglutinate microorganisms without antimicrobial activity as well as the specific interaction with Caco-2 cell surfaces, suggests molecular recognition as the primary role of this protein, targeting the invading agents and foreign substances as part of the immune response. Given the morphophysiological alterations observed in the small gut epithelium of rats fed with a PsSC-containing diet, we propose PsSC as an egg defense against predation through an anti-nutritive effect: the consumption of eggs would alter digestive mechanisms thus deterring predators. Similar effects were described in *P*. *canaliculata* eggs, which have a toxin in addition to lectins; in this species the eggs are brightly colored which would also act as a warning (aposematic) defense. In *P*. *scalaris* coloration is less bright, but, as both species overlap in geographical distribution, mimetic defense, through Müllerian mimicry, cannot be ruled out. The antinutritive mechanism present in the eggs of these snails seems a convergent evolution between plants and animals, as it is similar to the effects previously described for plant seed lectins [21,48,49].

## Conclusions

We provide evidence of a potentially new embryo defense mechanism in the genus *Pomacea* involving the lectin activity of the most abundant egg protein of *P*. *scalaris*, the carotenoprotein PsSC. Noteworthy, the primary structure of PsSC does not allow its inclusion to any known lectin family. The presence of a novel biological activity in PsSC amongst a group of carotenoproteins with conserved structure-function relationships expands our knowledge regarding the reproductive biology of these freshwater snails opening new avenues of research. For instance, it is not clear whether aposematism and/or mimicry are important defense mechanisms. More work is needed to shed light on the structure-function relationship of this snail lectin as well as on the role microbiota of a final egg predator could play on its anti-nutritive effect.

## Supporting information

S1 Table Full list of oligosaccharide structures recognized by PsSC. (XLS)
